# The cost of mentalizing: executive contributions to social cognition performance in amyotrophic lateral sclerosis

**DOI:** 10.3389/fpsyg.2026.1960658

**Published:** 2026-10-13

**Authors:** Chiara Cerami, Andrea Panzavolta, Veronica Faltracco, Eleonora Dalla Bella, Nilo Riva, Alessandra Telesca, Federica Ferrara, Alessandra Dodich, Christian Lunetta, Monica Consonni

**Affiliations:** 1 IUSS Cognitive Neuroscience (ICoN) Center, Scuola Universitaria Superiore IUSS, Pavia, Italy; 2 Istituti Clinici Scientifici Maugeri IRCCS, Brain e-Health Aging (BeA) Laboratory, Department of Neurorehabilitation, Milan, Italy; 3 Third Neurology Unit and Motor Neuron Disease Centre, Fondazione IRCCS Istituto Neurologico Carlo Besta, Milan, Italy; 4 Center for Mind/Brain Sciences (CIMeC), University of Trento, Rovereto, Italy

**Keywords:** amyotrophic lateral sclerosis, executive deficits, mediation analysis, mentalizing, social cognition

## Abstract

**Background:**

Mentalizing deficits are common in amyotrophic lateral sclerosis (ALS). Performance at high-level socio-cognitive tasks may be particularly vulnerable to integrity of basic cognitive processes. However, the extent to which non-social functioning contributes to socio-cognitive impairment in ALS remains poorly understood. In this study, we addressed this question in a large cohort of patients.

**Methods:**

We assessed 257 ALS patients and 120 healthy controls using the Story-based Empathy Task (SET), which dissociates intention attribution (IA), emotion attribution (EA), and basic causal inference (CI). Vectorial analyses were applied to quantify overall performance and imbalance between social and non-social components. Mediation analyses were conducted to evaluate the contribution of executive system integrity to social cognition performance in ALS.

**Results:**

Patients showed significantly reduced performance across all SET conditions compared to controls, with imbalance between mentalizing and basic causal inference. Distinct profiles emerged across ALS phenotypes. ALS patients with behavioral changes exhibited predominant deficits in emotion attribution, whereas cognitively unimpaired ALS showed selective impairment in intention attribution. Executive functioning was significantly associated with SET performances and mediated the relationship between basic inference and both intention and emotion attribution.

**Discussion:**

Mentalizing impairment in ALS reflects an interaction between executive processes and specialized socio-cognitive systems. The identification of dissociable profiles supports a dual-route, network-based model of ALS, involving socio-affective and inferential pathways, and underscores the importance of integrated cognitive assessment and more targeted interventions addressing both social and non-social cognitive domains.

## Introduction

Amyotrophic lateral sclerosis (ALS) is a multisystem disorder with motor and extra-motor involvement (Al-Khayri et al., 2024). At the diagnosis, a substantial proportion of individuals with ALS exhibit cognitive and/or behavioral impairments (Consonni et al., 2013; Rusina et al., 2021; De Marchi et al., 2025), meeting diagnostic criteria for frontotemporal dementia (FTD) in a consistent subset [i.e., 8–15% (Govaarts et al., 2016; Chalitsios et al., 2026)]. Cognitive and behavioral manifestations usually occur in the early phases of the disease independently of motor severity, supporting the view that ALS is a network-based disorder affecting distributed frontotemporal networks as well as motor systems (Cerami et al., 2015; Crespi et al., 2016; De Marchi et al., 2021; Poletti et al., 2024). Within this spectrum, socio-cognitive deficits, encompassing theory of mind (ToM), emotion recognition, empathy, and social behavioral changes (Henry et al., 2016), are increasingly recognized as a core component of the ALS cognitive phenotype (Cerami et al., 2014a; Crespi et al., 2020; Carelli et al., 2021; Palumbo et al., 2022; Faltracco et al., 2024).

Converging evidence suggests that mentalizing, namely the ability to attribute mental states to oneself and to others (Eikelboom et al., 2025), helping humans to successfully navigate the interpersonal world by making common sense explanations of behavior, is particularly impaired in ALS (Cerami et al., 2014a; Palumbo et al., 2022; Trojsi et al., 2017). These deficits have substantial clinical implications, especially on interpersonal communications, insight into illness, adherence to multidisciplinary care recommendations, and participation in complex decision-making processes, ultimately influencing quality of life and disease management outcomes (Crespi et al., 2020; Palumbo et al., 2022).

Performances at high-level socio-cognitive tasks may be particularly vulnerable to integrity of the executive system (Roca, 2016; Pagnoni et al., 2022). Inhibitory control deficits can compromise context-appropriate interpretations of mentalizing tasks as well as the suppression of salient but incorrect choices (Bull et al., 2008; Brass et al., 2009). Comparably, many other social cognition tasks based on complex verbal [e.g., false belief or faux-pas (Stone et al., 1998; Baron-Cohen et al., 1999)] or visual stimuli [e.g., TASIT (McDonald et al., 2003)] impose non-trivial demands on other executive sub-components (i.e., set shifting, working memory, planning and logic reasoning). As a result, reduced performance on mentalizing conditions may reflect, at least in part, global cognitive inefficiency (Palumbo et al., 2024). Disentangling these components is essential to interpret test results accurately and correctly plan care management and interventions in patients. In this context, the use of tasks capable of disentangling social from non-social cognitive components, and more ecological paradigms able to capture the complexity of social cognition domain, is crucial to understand the neurocognitive mechanisms underlying social dysfunctions and to improve research outcomes and clinical strategies.

To this end, the present study aimed to investigate social cognition performance in a large cohort of ALS patients by examining the extent to which mental state attribution relies on executive resources. Using a mentalizing paradigm [i.e., Story-based Empathy Task, SET (Dodich et al., 2015)], specifically designed to dissociate physical causality from intentional causality for mental and emotional states, we aimed to quantify potential imbalances between domain-general inferential processes and social inference, and determine whether executive functioning mediates the relationship between basic task performance and mental and emotional state attribution outcomes.

## Materials and methods

### Participants

A total of 377 individuals participated in the study with a 2:1 ratio between ALS patients (*n* = 257) and healthy control participants (HC, *n* = 120). Patients were consecutively enrolled at the Motor Neuron Diseases Centre of the ‘Carlo Besta’ Neurological Institute of Milan. Patients with first language other than Italian, clinical and imaging evidence of cerebrovascular disease, neurocognitive developmental disorder and primary psychiatric disorders were excluded. HCs were recruited at local community centers. Exclusion criteria included a positive history of neuropsychiatric disorders, pathological signs at the neurologic examination, Clinical Dementia Rating (CDR) global score >0, and a Mini-Mental State Examination raw score ≤26. None of the HC subjects were taking any medication interfering with neurobehavioral functioning. Each participant was enrolled after giving written informed consent. Participant’s consent was obtained according to the Declaration of Helsinki. The database was anonymized according to the Italian law for the protection of privacy. The study was approved by the local Ethic Committee of Fondazione IRCCS Istituto Neurologico Carlo Besta, Milan, Italy. Demographic and clinical characteristics of participants are shown on Table 1.

**Table 1:** Demographic and clinical features of the sample.

Variables	ALS	HC	Statistics
Sex *(male/female ratio)*	138/119	49/71	*X*^2^(1) = 5.41; *p* = **0.02**
Age in years *(mean ± st.dev.)*	61.4 ± 11.1	62.5 ± 8.3	*U* = 15,291; *p* = 0.8
Education in years *(mean ± st.dev.)*	11.9 ± 4.0	12.1 ± 4.2	*U* = 15,019; *p* = 0.6
Disease duration in months *(mean ± st.dev.)*	23.0 ± 23.7	—	—
ALS Strong phenotype	89 (34%) ALS-cu	—	—
	77 (30%) ALS-ci		
	38 (15%) ALS-bi		
	38 (15%) ALS-cbi		
	15 (6%) ALS-FTD		
MMSE raw score *(mean ± st.dev.)*	—	28.9 ± 0.9	—
	ALSFRS-r *(mean ± st.dev.)*	39.4 ± 5.6	—	—
ECAS total *(mean ± st.dev.)*	98.5 ± 18.9	—	—
ECAS ALS specific *(mean ± st.dev.)*	72.5 ± 15.4	—	—
ECAS ALS non-specific *(mean ± st.dev.)*	25.9 ± 5.0	—	—
ECAS language *(mean ± st.dev.)*	23.6 ± 3.4	—	—
ECAS fluency *(mean ± st.dev.)*	17.1 ± 5.5	—	—
ECAS executive functions *(mean ± st.dev.)*	31.8 ± 8.8	—	—
ECAS memory *(mean ± st.dev.)*	14.5 ± 4.5	—	—
ECAS visuo-spatial *(mean ± st.dev.)*	11.3 ± 1.0	—	—
Stroop time score *(mean ± st.dev.)*	22.2 ± 10.6	—	—
Stroop error score *(mean ± st.dev.)*	2.0 ± 3.9	—	—
FBI *(mean ± st.dev.)*	4.1 ± 5.2	—	—
HADS MND-total *(mean ± st.dev.)*	8.7 ± 5.5	—	—
HADS MND-A *(mean ± st.dev.)*	5.7 ± 3.6	—	—
HADS MND-D *(mean ± st.dev.)*	3.0 ± 2.7	—	—

ALS, amyotrophic lateral sclerosis; HC, healthy control subjects; MMSE, mini mental state examination; ALSFR-r, Amyotrophic Lateral Sclerosis Functional Rating Scale revised; ECAS, Edinburgh Cognitive Assessment Scale; FBI, frontal behavioral inventory; HADS MND-Total, Hospital Anxiety and Depression Scale for Motor Neuron Disease global score; HADS MND-A, Hospital Anxiety and Depression Scale for Motor Neuron Disease anxiety score; HADS MND-D, Hospital Anxiety and Depression Scale for Motor Neuron Disease depression score. Bold values indicates statistically significant difference.

### Clinical and neuropsychological assessment

Patients underwent a thorough clinical-neuropsychological assessment including Edinburgh Cognitive and Behavioral ALS Screen (ECAS) (Niven et al., 2015; Poletti et al., 2018), Stroop test (Caffarra et al., 2012), Frontal Behavioral Inventory (FBI) (Alberici et al., 2007), Hospital Anxiety and Depression Scale for Motor Neuron Disease (HADS-MND) (Faltracco et al., 2025) and the ALS Functional Rating Scale revised (ALSFRS-r) (Cedarbaum et al., 1999). Cognitive performance at the ECAS battery (Poletti et al., 2018) and behavioral assessment through the FBI questionnaire (Alberici et al., 2007) administered with a semi-structured caregiver interview were used to classify patients according to Strong’s criteria (Strong et al., 2017), as cognitively and behaviorally unimpaired (ALScu), cognitively impaired (ALSci), behaviorally impaired (ALSbi), cognitively and behaviorally impaired (ALScbi), or ALS with frontotemporal dementia (ALS-FTD).

### Social cognition assessment

All subjects were administered with a standardized nonverbal cartoon task, namely the SET (Dodich et al., 2015), consisting of two main experimental conditions (i.e., intention attribution—IA and emotion attribution—EA), plus a control condition entailing the comprehension of causality based on knowledge about the physical properties of objects or human bodies (i.e., causal inference—CI). The test lasts 15/20 min, each condition includes six trials and the subject’s task is to select the correct ending of a comic strip among three different possible choices. A global score (GS) of 18 indicates the best possible task performance. Each condition (i.e., IA, EA and CI) has a maximum score of 6 points. To help subjects to familiarize with the task, they performed a “trial” run, consisting of an example of causal attribution that would not appear in the testing phase.

### Statistical analyses

Descriptive statistics were calculated, including frequency and percentage for categorical variables, mean and standard deviation for pseudo-continuous variables. Dependent measures were preliminary analyzed to test for normality and heteroscedasticity. Since some demographic, clinical and neuropsychological variables did not have a normal distribution, the Mann–Whitney *U* test was used for group comparisons. Sex distribution was evaluated through Chi square test.

A vectorial analysis (Dodich et al., 2016) was performed to calculate the overall performance (*d*) and the balance (*α*) between attribution of intentions and emotions (IA & EA conditions) and basic cognitive demand (CI condition) at SET. The *d*-values were obtained computing the distance in a two-dimensional Cartesian space between a point with the coordinates (SET-CI, SET-IA/EA) and the origin, while the *α*-values through inverse trigonometric functions. Comparisons between the indices of vectorial analyses have been conducted in the whole ALS group vs. HC, as well as in each Strong ALS subgroup (Strong et al., 2017) vs. HC group, using the Mann–Whitney test. Then, we explored the correlation (Spearman’s rank correlation coefficient) between executive function ability, mood changes and SET performances.

Based on correlation results, a mediation analysis was used to assess the weight of executive function ability on SET performances. Stroop Error was selected as the executive measure for the analysis because, unlike the ECAS executive subscore used for cognitive phenotyping, it provided an independent index of inhibitory control. Moreover, it was less likely than Stroop Time to be confounded by motor or speech-related slowing in ALS (Amato et al., 2013; Collette et al., 2007). In detail, we conducted two mediation analyses to assess the indirect effect of executive function ability (i.e., Stroop Error score) on the relationship between basic cognitive functioning as measured by SET-CI condition and the two socio-cognitive conditions (SET-IA & SET-EA). For each model, the standardized coefficients and the statistical significance of the total, direct and indirect (mediated) effects were analyzed using the multilevel mediation (MLM) estimator with 5,000 bootstraps samples. Statistical analyses were computed though JASP vers. 0.19.1.

## Results

### Group comparisons and vectorial analyses

Descriptive statistics of demographic, clinical, and cognitive variables are illustrated in Table 1. Based on the cognitive assessment and according to Strong’s criteria, one third of the sample was classified as ALS-cu (i.e., 34%, 89 out of 257), whereas the remaining ALS individuals exhibited impairments in cognition or behavior (i.e., ALSci = 30%, ALSbi = 15%, ALScbi = 15%, ALS-FTD = 5%).

Mann–Whitney analysis on SET performances highlighted significant differences between the whole ALS group and HCs in all SET sub-conditions (Table 2). ALS also showed significantly lower dSET-IA and dSET-EA values with respect to HC, and a significantly higher αSET-IA value (imbalance between SET-IA and SET-CI). Looking at the ALS neuropsychological phenotypes, lower SET-IA, SET-EA and SET-CI scores were found in ALSci, ALScbi and ALS-FTD compared to HCs, while ALScu showed lower scores only in SET-IA and ALSbi in SET-EA. ALScu showed also significantly lower αSET-IA values compared to HCs, while ALScbi lower αSET-IA and αSET-EA. Finally, ALSci, ALScbi, and ALS-FTD subgroups showed significantly lower dSET-IA and dSET-EA values compared to HCs. See Supplementary Tables S1–S5 for details on comparison analyses at the ALS subgroup level.

**Table 2:** Comparisons between the whole ALS group and HCs at SET scores and vectorial indices.

Variables	ALS	HC	Statistics
SET-GS raw score	12.9 ± 3.6	14.7 ± 3.0	*U* = 10,501; *p* < **0.001**
SET-IA raw score	4.3 ± 1.5	5.1 ± 1.1	*U* = 10,541; *p* < **0.001**
SET-CI raw score	4.4 ± 1.3	4.7 ± 1.2	*U* = 13,433; *p* = **0.03**
SET-EA raw score	4.2 ± 1.3	4.9 ± 1.2	*U* = 10,923; *p* < **0.001**
d_SET-IA_	6.2 ± 1.6	7.0 ± 1.3	*U* = 11,232; *p* < **0.001**
d_SET-EA_	6.2 ± 1.6	6.9 ± 1.5	*U* = 11,354; *p* < **0.001**
α_SET-IA_	43.8 ± 13.0	47.5 ± 9.3	*U* = 12,927; *p* = **0.01**
α_SET-EA_	43.8 ± 12.7	46.4 ± 8.0	*U* = 13,552; *p* = 0.05

ALS, amyotrophic lateral sclerosis; HC, healthy control subjects; SET, story-based empathy task; SET-GS: global score; SET-IA: intention attribution; SET-CI: causal attribution; SET-EA: emotion attribution. Bold values indicates statistically significant difference.

### Correlation and mediation analyses

Correlation analyses assessing the relationship between executive functioning, mood changes, and SET performances revealed significant negative associations between Stroop indices and both SET-GS and sub-condition scores. Stroop Time was correlated with SET-GS (*r* = −0.380, *p* < 0.001), SET-IA (*r* = −0.315, *p* < 0.001), SET-CI (*r* = −0.283, *p* < 0.001), and SET-EA (*r* = −0.298, *p* < 0.001), Stroop Error showed similar associations (SET-GS: *r* = −0.397, *p* < 0.001; SET-IA: *r* = −0.344, *p* < 0.001; SET-CI: *r* = −0.319, *p* < 0.001; SET-EA: *r* = −0.308, *p* < 0.001). No other significant correlations were observed.

On this basis, we selected the Stroop Error score as measure of executive function to test two mediation models. The first mediation model including SET-IA as dependent variable highlighted a strong significant direct effect (*β* = 0.282, *p* < 0.001) between basic cognitive demands (SET-CI) and intention attribution (SET-IA), as well as a significant indirect effect of executive function performance (*β* = 0.064; *p* = 0.006) mediating approximately 18% of the total effect. The same pattern has been found in the second mediation model with SET-EA as dependent variable showing a strong direct effect (*β* = 0.279, *p* < 0.001) and a significant indirect effect of the Stroop Error score (*β* = 0.074, *p* = 0.02) mediating approximately 21% of the total effect.

See Figure 1 and Supplementary Table S6 for details on mediation analyses.

**Figure 1:**
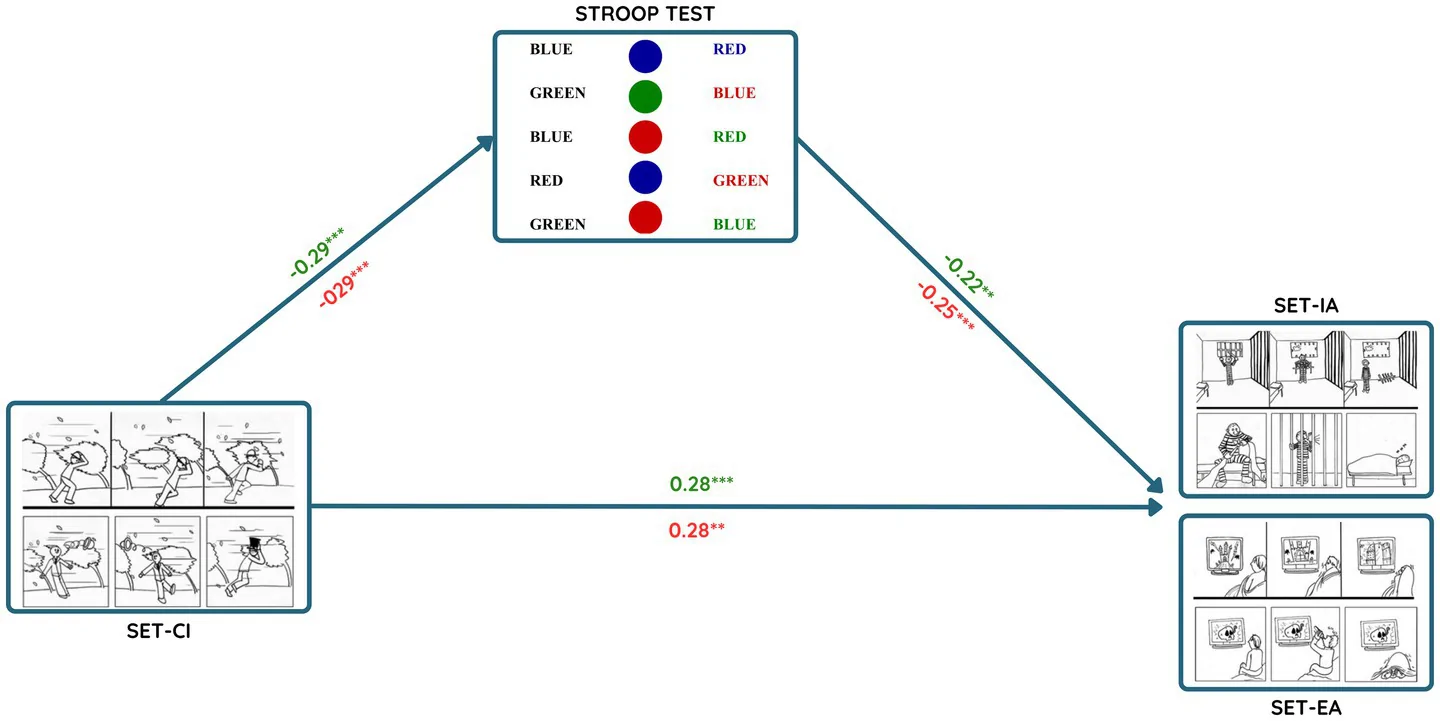
Mediation models tested SET-IA (Model 1) and SET-EA (Model 2). Numbers on arrows represent standardized estimated path coefficients (Model 1, in green; Model 2, in red). SET stimuli reproduced from Dodich et al. (2015), with permission from Springer Nature. * = *p* < 0.05; ** = *p* < 0.01; *** = *p* < 0.001.

## Discussion

The present study provides converging evidence that social cognition impairment in ALS is closely linked to other cognitive processes. Alterations in mental state and emotion attribution co-occur with deficits in basic inferential processing, supporting a model in which social and non-social cognition share partially overlapping neurocognitive substrates. This interdependence is further reinforced by mediation results, indicating that executive functioning significantly contributes to the relationship between basic inferential abilities and social cognition outcomes. Notably, mood symptoms did not significantly contribute to mentalizing outcomes in the present study. However, this finding should be interpreted with caution, as the restricted range of mood disturbances explored by study measures may have limited our ability to detect significant associations in our sample.

Group comparisons and vectorial analyses identify two partially dissociable socio-cognitive profiles within the ALS spectrum. Beyond conventional scoring, vectorial approach highlights performance profiles by capturing the balance (or imbalance) between performance on mentalizing conditions and basic cognitive demands. Such metrics may be informative in ALS, where subtle dissociations (e.g., relatively preserved control performance with disproportionately reduced intention/emotion attribution) could emerge. In our sample, patients with frontotemporal phenotypes (ALSbi, ALScbi, ALS-FTD) showed predominant impairments in emotion attribution, consistent with the socio-affective deficits described in FTD and linked to degeneration of fronto-limbic regions, including orbitofrontal cortex, anterior cingulate cortex, and anterior insula (Kumfor and Piguet, 2012; Cerami et al., 2014a, 2014b; Crespi et al., 2016). These findings further support the conceptualization of ALS as part of the frontotemporal lobar degeneration spectrum (Strong et al., 2017). On the contrary, cognitively unimpaired ALS patients showed isolated disruption of inferential mechanisms supporting mental state reasoning. This pattern can be interpreted within the framework of embodied cognition and motor simulation theory, which theorizes that understanding others’ actions and intentions relies on internally simulating others’ behavior (Gallese and Goldman, 1998), mediated by the mirror neuron system (Rizzolatti et al., 2001; Cattaneo and Rizzolatti, 2009). Observed actions are first mapped onto motor representations and then integrated within higher-order mentalizing regions to support intention inference (Rizzolatti et al., 2001; Keysers and Gazzola, 2007). In ALS, neurodegeneration extends far beyond the primary motor cortex to include premotor and frontoparietal regions, part of action representation and prediction networks (De Marchi et al., 2025). This may result in impaired mapping between observed actions and internal models, ultimately affecting intention attribution. Unlike intention understanding, emotion inference is less dependent on the integrity of motor representations and more grounded in the mapping of others’ emotional states onto internal visceral and affective representations mediated by fronto-limbic and salience networks, including the anterior insula, anterior cingulate cortex, and orbitofrontal cortex (Adolphs, 2002; Keysers and Gazzola, 2007; Critchley and Harrison, 2013; Uddin, 2015). Overall, these findings align with a dual-route model of social cognition impairment in ALS, involving a socio-affective pathway (fronto-limbic) and an inferential pathway (motor–premotor and parietal systems) (Shamay-Tsoory and Aharon-Peretz, 2007). While deficits in intention attribution in ALS reflect disruption of action–simulation mechanisms linked to the core degeneration of sensorimotor system (Jelsone-Swain et al., 2015), alterations in emotion attribution are primarily associated with fronto-limbic pathology, particularly in those neurophenotypes belonging to the ALS–FTD continuum (Savage et al., 2014; Crespi et al., 2016; Strong et al., 2017).

Executive functioning significantly mediated the relationship between basic inferential abilities and social cognition performance, supporting the notion of a cognitive cost for higher-order social cognition tasks. This is in line with recent theoretical models emphasizing that social cognition emerges from the interaction between domain-specific and domain-general processes (Eikelboom et al., 2025). Mentalizing recruits many processes (perception, attention, memory, motivation) through social-specific content, and core inferential processing is highly computational and demanding (Schaafsma et al., 2015). Results of mediating analysis showed that in ALS inhibitory control deficits, as measured by Stroop Error score, may act as key limiting factors for efficient inference attribution. However, the persistence of a direct effect indicates that domain-specific socio-cognitive impairments remain independently relevant, according to current models of ToM (Eikelboom et al., 2025).

Some limitations of the study should be acknowledged. Its cross-sectional design precludes conclusions about the longitudinal evolution of distinct patterns of inferential and socio-cognitive impairment in ALS. Moreover, executive functioning was primarily assessed through inhibitory control, and future studies incorporating broader measures of executive processes should be recommended to quantify the weight of the different executive subcomponents on socio-cognitive processing, as well as to explore whether the observed relationships show similar patterns in healthy individuals. Despite these limitations, our results offer a novel framework to account for socio-cognitive heterogeneity in ALS.

Overall, study findings support a complex dual network-based model of socio-cognitive impairments in ALS and underscore the importance of integrated neuropsychological assessment approaches targeting both socio-cognitive and non-social sub-domains. From a translational perspective, given the impact of such cognitive deficits on communication, interpersonal relationships and decision-making capacity, early and better identification may support more targeted interventions, including cognitive rehabilitation, caregiver education and support strategies, and personalized clinical management. Future studies involving large phenotype-stratified ALS cohorts and integrating neuropathological evidence should thus be promoted to better inform ALS researchers and clinicians opening the way to targeted mechanism-based cognitive interventions.

## Data Availability

The raw data supporting the conclusions of this article will be made available by the authors, without undue reservation.
