# Supplementary material for: The cost of mentalizing: executive contributions to social cognition performance in amyotrophic lateral sclerosis
Source: Front Psychol. 2026 Oct 13;17:1960658. doi: 10.3389/fpsyg.2026.1960658 (PMC13710097; doi:10.3389/fpsyg.2026.1960658)
Supplement: Supplementary file 1 [file Table_1.docx]

**Supplementary Materials**

**Supplementary Table S1.** Comparison between cognitively unimpaired ALS (ALScu) and HC at SET and vectorial indices

|  | **ALScu** | **HC** | **Statistics** |
| --- | --- | --- | --- |
| SET-GS raw score | 15.0 ± 2.1 | 14.7 ± 3.0 | U= 5214; p=0.7 |
| SET-IA raw score | 5.0 ± 1.0 | 5.1 ± 1.1 | U= 5032; p=0.4 |
| SET-CI raw score | 5.0 ± 0.8 | 4.7 ± 1.2 | U= 4801; p= 0.19 |
| SET-EA raw score | 5.0 ± 1.0 | 4.9 ± 1.2 | U= 5209; p=0.7 |
| d_SET-IA_ | 7.1 ± 1.0 | 7.0 ± 1.3 | U= 5255; p= 0.8 |
| d_SET-EA_ | 7.1 ± 0.9 | 6.9 ± 1.5 | U= 5190; p= 0.7 |
| α_SET-IA_ | 44.7 ± 6.8 | 47.5 ± 9.3 | U= 4397; **p= 0.02** |
| α_SET-EA_ | 44.7 ± 7.4 | 46.5 ± 8.0 | U= 4697; p= 0.1 |
| *SET: Story-based Empathy Task; SET-GS: Global Score; SET-IA: Intention Attribution;*  *SET-CI: Causal Attribution; SET-EA: Emotion Attribution* | | | |

**Supplementary Table S2.** Comparison between the ALSci and HC at SET and vectorial indices

|  | **ALSci** | **HC** | **Statistics** |
| --- | --- | --- | --- |
| SET-GS raw score | 11.3 ± 3.9 | 14.7 ± 3.0 | U= 2208; **p< 0.001** |
| SET-IA raw score | 3.6 ± 1.6 | 5.1 ± 1.1 | U= 2222**; p< 0.001** |
| SET-CI raw score | 3.8 ± 1.6 | 4.7 ± 1.2 | U= 3120**; p= 0.03** |
| SET-EA raw score | 3.8 ± 1.6 | 4.9 ± 1.2 | U= 2682**; p< 0.001** |
| d_SET-IA_ | 5.5 ± 1.8 | 7.0 ± 1.3 | U= 2316; **p< 0.001** |
| d_SET-EA_ | 5.5 ± 1.9 | 6.9 ± 1.5 | U= 2594; **p< 0.001** |
| α_SET-IA_ | 43.6 ± 17.8 | 47.5 ± 9.3 | U= 4070; p= 0.1 |
| α_SET-EA_ | 45.4 ± 16.2 | 46.5 ± 8.0 | U= 4531; p= 0.8 |
| *SET: Story-based Empathy Task; SET-GS: Global Score; SET-IA: Intention Attribution;*  *SET-CI: Causal Attribution; SET-EA: Emotion Attribution* | | | |

**Supplementary Table S3.** Comparison between the ALSbi and HC at SET and vectorial indices

|  | **ALSbi** | **HC** | **Statistics** |
| --- | --- | --- | --- |
| SET-GS raw score | 14.3 ± 2.4 | 14.7 ± 3.0 | U= 1913; p=0.1 |
| SET-IA raw score | 4.7 ± 1.2 | 5.1 ± 1.1 | U= 1890; p=0.09 |
| SET-CI raw score | 4.8 ± 1.0 | 4.7 ± 1.2 | U= 2190; p= 0.7 |
| SET-EA raw score | 4.6 ± 1.0 | 4.9 ± 1.2 | U= 1790**; p=0.03** |
| d_SET-IA_ | 6.9 ± 1.1 | 7.0 ± 1.3 | U= 2041; p= 0.3 |
| d_SET-EA_ | 6.8 ± 1.1 | 6.9 ± 1.5 | U= 1967; p= 0.1 |
| α_SET-IA_ | 44.3 ± 10.9 | 47.5 ± 9.3 | U= 1909; p= 0.1 |
| α_SET-EA_ | 43.9 ± 8.7 | 46.5 ± 8.0 | U= 1875; p= 0.09 |
| *SET: Story-based Empathy Task; SET-GS: Global Score; SET-IA: Intention Attribution;*  *SET-CI: Causal Attribution; SET-EA: Emotion Attribution* | | | |

**Supplementary Table S4.** Comparison between the ALScbi and HC at SET and vectorial indices

|  | **ALScbi** | **HC** | **Statistics** |
| --- | --- | --- | --- |
| SET-GS raw score | 11.3 ± 3.2 | 14.7 ± 3.0 | U= 931; **p< 0.001** |
| SET-IA raw score | 3.6 ± 1.5 | 5.1 ± 1.1 | U= 1030**; p< 0.001** |
| SET-CI raw score | 4.1 ± 1.2 | 4.7 ± 1.2 | U= 1689**; p= 0.01** |
| SET-EA raw score | 3.4 ± 1.3 | 4.9 ± 1.2 | U= 925**; p< 0.001** |
| d_SET-IA_ | 5.7 ± 1.5 | 7.0 ± 1.3 | U= 1160; **p< 0.001** |
| d_SET-EA_ | 5.5 ± 1.4 | 6.9 ± 1.5 | U= 1050; **p< 0.001** |
| α_SET-IA_ | 40.7 ± 12.9 | 47.5 ± 9.3 | U= 1588; **p= 0.004** |
| α_SET-EA_ | 39.9 ± 12.9 | 46.5 ± 8.0 | U= 1638; **p= 0.007** |
| *SET: Story-based Empathy Task; SET-GS: Global Score; SET-IA: Intention Attribution;*  *SET-CI: Causal Attribution; SET-EA: Emotion Attribution* | | | |

**Supplementary Table S5.** Comparison between the ALS-FTD and HC at SET and vectorial indices

|  | **ALS-FTD** | **HC** | **Statistics** |
| --- | --- | --- | --- |
| SET-GS raw score | 9.4 ± 3.6 | 14.7 ± 3.0 | U= 236; **p< 0.001** |
| SET-IA raw score | 3.4 ± 1.6 | 5.1 ± 1.1 | U= 368**; p< 0.001** |
| SET-CI raw score | 3.2 ± 1.4 | 4.7 ± 1.2 | U= 375**; p< 0.001** |
| SET-EA raw score | 2.8 ± 1.7 | 4.9 ± 1.2 | U= 319**; p< 0.001** |
| d_SET-IA_ | 4.8 ± 1.7 | 7.0 ± 1.3 | U= 291; **p<0.001** |
| d_SET-EA_ | 4.5 ± 1.6 | 6.9 ± 1.5 | U= 254; **p<0.001** |
| α_SET-IA_ | 46.9 ± 16.7 | 47.5 ± 9.3 | U= 836; p=0.9 |
| α_SET-EA_ | 40.6 ± 21.1 | 46.5 ± 8.0 | U= 811; p=0.5 |
| *SET: Story-based Empathy Task; SET-GS: Global Score; SET-IA: Intention Attribution;*  *SET-CI: Causal Attribution; SET-EA: Emotion Attribution* | | | |

**Supplementary Table S6.** Results of two mediation analyses examining the direct, indirect, and total effects of basic cognitive functioning (SET-CI) on socio-cognitive abilities (SET-IA and SET-EA), with executive function ability (Stroop Error score) as mediator. Model 1 tests the mediation pathway between SET-CI and intentional attribution (SET-IA); Model 2 tests the mediation pathway between SET-CI and emotional attribution (SET-EA). Effects are reported as standardized coefficients with 95% Confidence Intervals (CI).

| ***Mediation***  ***models*** | ***Direct effect***  ***(95% CI)*** | ***Indirect effect***  ***(95% CI)*** | ***Total effect***  ***(95% CI)*** |
| --- | --- | --- | --- |
| *Model 1 – SET-IA* | 0.282***  (0.190 to 0.366) | 0.064**  (0.019 to 0.146) | 0.346***  (0.271 to 0.423) |
| *Model 2 – SET-EA* | 0.279***  (0.195 to 0.353) | 0.074**  (0.038 to 0.130) | 0.353***  (0.279 to 0.421) |
| SET-IA: Story-based Empathy Task Intention Attribution; SET-IA: Story-based Empathy Task Emotion Attribution; CI: Confidence Interval. * = p < 0.05; ** = p < 0.01; *** = p < 0.001 | | | |
